# Sentinel lymph node biopsy is unsuitable for routine practice in younger female patients with unilateral low-risk papillary thyroid carcinoma

**DOI:** 10.1186/1471-2407-11-386

**Published:** 2011-09-02

**Authors:** Ou Huang, WeiLi Wu, OuChen Wang, Jie You, Quan Li, DuPing Huang, XiaoQu Hu, JinMiao Qu, Cun Jin, YouQun Xiang, Kai Yang, ShuMei Zhou, XueMin Chen, YiFei Pan, GuiLong Guo, XiaoHua Zhang

**Affiliations:** 1 Department of Surgery, Ruijin Hospital, School of Medicine, Shanghai Jiaotong University, Shanghai 200025, China; 2 Department of Surgical Oncology, the Third Affiliated Hospital of Wenzhou Medical College, Wenzhou 325200, Zhejiang Province, China; 3 Department of Surgical Oncology, the First Affiliated Hospital of Wenzhou Medical College, Wenzhou 325000, Zhejiang Province, China

## Abstract

**Background:**

Sentinel lymph node (SLN) biopsy has been used to assess patients with papillary thyroid carcinoma (PTC). To achieve its full potential the rate of SLN identification must be as close to 100 percent as possible. In the present study we compared the combination of preoperative lymphoscintigraphy scanning by sulfur colloid labeled with 99 m Technetium, gamma-probe guided surgery, and methylene blue with methylene blue, alone, for sentinel node identification in younger women with unilateral low-risk PTC.

**Methods:**

From January 2004 to January 2007, 90 female patients, ages 23 to 44 (mean = 35), with unilateral low-risk PTC (T_1-2_N_0_M_0_) were prospectively studied. Mean tumor size was 1.3 cm (range, 0.8-3.7 cm). All patients underwent unilateral modified neck dissection. Prior to surgery, patients had, by random assignment, identification and biopsy of SLNs by methylene blue, alone (Group 1), or by sulfur colloid labeled with 99 m Technetium, gamma-probe guided surgery and methylene blue (Group 2).

**Results:**

In the methylene blue group, SLNs were identified in 39 of 45 patients (86.7%). Of the 39 patients, 28 (71.8%) had positive cervical lymph nodes (pN+), and 21 patients (53.8%) had pSLN+. In 7 of the 28 pN+ patients (25%), metastases were also detected in non-SLN, thus giving a false-negative rate (FNR of 38.9% (7/18), a negative predictive value (NPV) of 61.1% (11/18), and an accuracy of 82.1% (32/39). In the combined technique group, the identification rate (IR) of SLN was 100% (45/45). Of the 45 patients, 27 (60.0%) had pN+, 24 (53.3%) had pSLN+. There was a FNR of 14.3% (3/21), a NPV of 85.7% (18/21), and an accuracy of 93.3% (42/45). The combined techniques group was significantly superior to the methylene blue group in IR (p = 0.035). There were no significant differences between two groups in sensitivity, specificity, NPV, or accuracy. Location of pN+ (55 patients) in 84 patients was: level I and V, no patients; level II, 1 patient (1.2%); level III, 6 patients (7.2%); level III and IV, 8 patients (9.5%); level IV, alone, 8 patients (9.5%); level VI, 32 patients (38.1%). In all 90 patients, IR of SLN was 93.3%, FNR, 25.6%, NPV, 74.4%, and accuracy rate, 88.1 percent.

**Conclusions:**

Compared to a single technique, there was a significantly higher SLN identification rate for the combined technique in younger female with ipsilateral, low-risk PTC (T_1-2_N_0_M_0_). Thus, a combined SLN biopsy technique seems to more accurately stage lymph nodes, with better identification of SLN located out of the central compartment. Regardless of the procedure used, the high FNR renders the current SLN techniques unsuitable for routine practice. Based on these results, prophylactic node dissection of level VI might be considered because 38.1% of our patients had such node metastases.

## Background

Nodal metastases are a significant risk factor for survival in patients with papillary thyroid cancer (PTC) [1]. Recent studies have suggested the use of sentinel lymph node (SLN) detection as a less invasive method for studying lymphatic metastatic spread in patients with breast [2,3], melanoma [4], cervical [5,6], and thyroid cancers [7,8]. SLN biopsy has potential advantages, for example, in reducing morbidity compared with elective lymph node dissection, and could offer a standardized technique of lymph node evaluation in PTC patients. Because of its high sensitivity, accuracy and low false-negative rate in predicting axillary lymph node (ALN) status, SLN biopsy has obtained widespread consensus and replaced routine ALN dissection as the standard for staging in breast cancer [9]. The success of SLN biopsy in breast cancer has set a good example for other types of solid tumors [3,4,6], including thyroid cancer [7,8]. It has been demonstrated that SLN biopsy for thyroid cancer is both feasible and safe, using intraoperative isosulfan blue vital dye or preoperative lymphoscintigraphy scanning [7,8]. However, there are differences between thyroid and breast cancer, so that questions need be answered before the utility of SLN biopsy becomes standard practice. For example, while lymphatic drainage is largely orderly progression in breast cancer, it is not as predictable in thyroid cancer, perhaps due to prior thyroid disease. Thus, it has been suggested that there be further investigation [7]. Whether SLN biopsy in thyroid cancer has a predictive value similar to that in breast cancer, thus replacing neck dissection, is needs to be confirmed.

Most studies concerning the SLN biopsy in thyroid cancer have been conducted in Western populations [7,8,10-20], while similar data is not available for the Chinese population. Moreover, the means used to identify the SLN varies. Some rely on intraoperative of blue dye (isosulfan blue dye, Canada)[7], patent blue dye [Patent Blue-V, Hong Kong) [17], methylene blue [Japan 13]), radiotracer (^99 m^Tc-colloid, Turkey) [21], ^99 m^Tc sulfur colloid (Switzerland) [19], and ^99 m^Tc nanocolloid (Italy) [19], to identify sentinel nodes. However, others rely on blue dye, alone. Furthermore, the risk of nodal metastases varies considerably between age and tumor size [22]. Age is the most important prognostic factor for thyroid cancer. According to the National Thyroid Cancer Treatment Cooperative Study (NTCTCS) staging classification registration, patients older than 45 with cervical lymph node metastases were classified as stage III (high risk), whereas those younger than 45 with such lymph node metastases were considered as stage I (low risk) [22]. The comparative analyses of SLN biopsy by methylene blue, only, or the combined technique have not been reported in younger-than-45 female patients with ipsilateral, low-risk PTC (T_1-2_N_0_M_0_) in either the Western or Chinese populations. Doing so could aid in determining the use of SLN biopsy in this PTC group.

For the above reasons, we hypothesized that SLN biopsy could provide accurate assessment of cervical lymph nodes, as it does in breast cancer, and initiated a clinical trial to define its role. By using intraoperative methylene blue, only, or the combined techniques (preoperative lymphoscintigraphy scanning by sulfur colloid labeled with ^99 m ^Technetium, gamma-probe guided surgery, and intraoperative blue dye injection). Further, we sought to identify which technique might have a better predictive value.

## Methods

### Patients

The study was approved by the institutional review board of Wenzhou Medical College, and informed consent was obtained in writing from all patients after discussion of risks and benefits with the operating surgeon. From January 2004 to January 2007, all patients with a preoperative diagnosis of PTC by fine-needle aspiration, clinically node-negative detected by clinical examination and ultrasound were asked to participate in this study. All were female and had only one unilateral tumor site by ultrasound. Exclusion criteria were a previous history of neck operation, current pregnancy, and known hypersensitivity to ethylene blue, age younger than 15 years or older than 44 years at diagnosis, tumor more than 4 cm in greatest dimension and limited to the thyroid, or extending beyond the thyroid under ultrasound, or tumor in isthmus. Preoperative evaluation included history, complete physical examination, routine laboratory evaluation, including serum TSH measurement, ultrasound of thyroid and neck, including ipsilateral cervical lymph nodes, and chest x-ray. All patients underwent attempted SLN biopsy, followed by unilateral modified radical neck dissection.

### SLN Biopsy

All surgeons had performed more than 20 SLN biopsies prior to the study, and had experience with thyroid SLN procedures. The day prior to the operation, lymphoscintigraphy was performed only for patients randomized to the combined procedure group. A single dose of 99 mTc sulfur colloid (~0.5 ml) was injected in the primary tumor for lymphoscintigraphy and intraoperative lymph node detection. Ten to 15 minutes after injection, dynamic images were detected by single photon emission computed tomography (PHILIPS Vertex V60, USA) to visualize lymphatic drainage. The position of the sentinel node was marked on the skin overlying the "hot spots" [23]. The operation was performed within 24 h after lymphoscintigraphy. After induction anesthesia, a standard transverse cervical incision was made, cutaneous flaps developed, and strap muscles retracted laterally. The thyroid lobe containing the tumor was exposed, dividing the medial thyroid vein and the upper pedicle. Both parathyroid glands were identified. Approximately 1.0 mL of 1% methylene blue was administered into the primary tumor with a 26-gauge needle. Care was taken not to stain surrounding tissue with blue dye. The thyroid gland was replaced in its normal position, and gentle massage was applied for 3 minutes. A combination of lymphoscintigraphy, blue lymph vessel and blue node identification, and a hand-held probe were used for tracing to the SLN. An SLN was defined as any blue node, or any node that could be identified as substantially radioactive above background activity. A specific SLN-to-background ratio was more than two for defining an SLN [24]. After the first radioactive SLN was removed, any node containing radioactive counts ≥ 10% of the *ex vivo *count of the most radioactive SLN was considered to be an additional SLN. The SLN was excised and radioactivity measured *ex vivo *to confirm nodal activity. After the SLN was removed, a central lymphadenectomy, unilateral total lobectomy and modified radical neck dissection were completed. Lymph nodes, including SLN, were sectioned along the long axis into two sections and then submitted for routine hematoxylin-eosin (H-E) staining. Each tissue block was sectioned serially. Location of positive lymph nodes (pN+) was recorded according to the Neck Dissection Classification update (revisions proposed by American Head and Neck Society and American Academy of Otolaryngology-Head and Neck Surgery [25]).

### Statistical Analysis

Results of SLN biopsy were quantified using the following definitions: accuracy = (true positive + true negative)/(total patients); sensitivity = (true positive)/(true positive + false negative); and specificity = (true negative)/(true negative + false positive). All data were statistically analyzed using chi-squared test. Fisher's exact test was used for comparison, if necessary. All statistical tests were two-sided and P < 0.05 was considered significant. All statistical analyses were performed with SPSS statistical software package 15.0.

## Results

Between January 2004 to January 2007, 90 patients were enrolled and randomly divided into two groups. Mean age was 35.1 years (range, 23-44 years). Mean tumor size was 1.3 cm (range, 0.8-3.7 cm). Fifty-two percent of tumors were localized to the right lobe, 48% to the left. The incidence of positive lymph nodes was 65.5%, successful identification of SLN 93.3%, false-negative rate 25.6%, and accuracy 88.1% (Table 1). SLN biopsy was performed using single-agent injection (methylene blue dye) in 45 and dual-agent injection (blue dye plus radioactive colloid) in 45 patients, respectively.

**Table 1:** Sentinel lymph node biopsy results

	Number of patients (%)		
	**methylene blue (n = 45)**	**dual-agent (n = 45)**	**In total (n = 84)**

Overall success in identification of SLN	39 (86.7%)	45 (100%)	84 (93.3%)
Mean No. of SLN in identified (range)	2.7 (1-7)	5.4 (3-10)	4.1
No. of patients with positive SLN	21 (53.8%)	24 (53.3%)	45 (53.6%)
No. of patients with negative SLN	18 (46.2%)	21 (46.7%)	39 (46.4%)
Mean No. of lymph nodes examined	21.3	22.4	21.9
No. of patients with positive lymph nodes	28(71.8%)	27(60.0%)	55 (65.5%)
Positive lymph nodes only in NSLN	7(25.0%)	3(11.1%)	10 (20.0%)
False negative rate	38.9%	14.3%	25.6%
Accuracy	82.1%	93.3%	88.1%

Comparison of the results using single- or dual-agent injection is listed in Table 1. In the methylene blue group, SLNs were identified in 39 of 45 patients (success rate = 86.7%). Of the 39, 28 patients (71.8%) had positive cervical lymph nodes (pN+). Of the 28 with positive cervical nodes, 7(25%) had metastases detected only in non-positive SLNs, thus giving a false-negative rate of 38.9%, a negative predictive value of 61.1% (11/18), and an accuracy rate of 82.1% (32/39).

In the combined technique group, SLN detection rate was 100% (45/45), with 27 of 45 patients (60.0%) having pN+, thus giving a false-negative rate of 14.3%, a negative predictive value of 85.7% (18/21), and an accuracy of 93.3% (42/45) [Table 1].

No positive lymph nodes in levels I or V were identified in the 84 patients. In one patient (1.2%), the pN was located in level II, in 6 patients (7.2%) in level III, in 8 patients (9.5%) in levels III and IV, in 8 patients (9.5%) in level IV, and in 32 patients (38.1%) in level VI.

## Discussion

Recent developments in SLN concept and technology have resulted in the application of this approach to define the first draining site or SLN to which a cancer may have metastasized [26,27]. The underlying thesis in solid tumor biology is that metastasis generally begins with an orderly progression, spreading through the lymphatic channels to the SLN that should be reflected in the pathological status of the subsequent lymph nodes. Thus, the purpose of a SLN biopsy is not to improve survival, since that has not been clearly demonstrated, but to obtain diagnostic and prognostic information in order to help select systemic therapy. With the possibility of systemic disease, prognosis can only be improved by the addition of adjuvant therapy. To some extent the degree of possibility is determined by the presence or absence of regional nodal involvement.

The SLN technique is currently mainly used in surgery for breast cancer and melanoma, although its use is increasing in other solid tumors [3,4,6], including PTC [7,8]. The SLN procedure has proven to be a valuable surgical adjunct and has become the standard for the surgical approach in the axillary staging of early-stage breast cancer, helping avoid unnecessary dissection in up to 75% of early-stage breast cancers [9]. Adjuvant chemotherapy regimen and whether adjuvant radiotherapy should or should not be given are related to the number of ALN involved [28]. Thus, accurate assessment of ALN status by ALN dissection is important, not only for staging and prognosis, but also for guiding treatment selection, when SLN biopsy is positive [29].

Different types of solid tumors have different patterns and biology of lymph node metastases [30]. In most other solid tumors, if the recurrence risk is high, more aggressive treatment may be warranted because the absolute reduction in the risk of recurrence is larger, thus offsetting disadvantages related to more aggressive treatment and improving the benefit-risk ratio. However, in the case of PTC, the generally favorable disease biology seems to make the extent of operation performed less important. PTC in younger "low risk" patients have a pattern of frequent (75%) regional nodal metastases when nodal resections are performed, but uncommonly (< 3%) have distant metastases, which are almost entirely confined to the lung when they occur, and a 99% disease-free survival rate at 20 years after treatment [31]. This unique clinical situation of very frequent nodal metastases but excellent survival suggests the use of SLN examination in younger patients with low-risk PTC [32]. Presently, as to the surgical options for PTC with gross lymph nodes involvement, there is no debate about the need for a radical neck dissection for prevention of local recurrence and systemic metastases [33]. The current controversy in the management of occult lymph node metastases in PTC revolves around the clinical contrast between the rather high incidence of metastases in patients with clinically occult nodes (50-80%), with a neck recurrence rate ranging from 1.4 to 30%, yet a 5-year mortality rate ranging from 0.9 to 17% when routine nodal dissection is not performed [34,35]. Moreover, the greatest complications occurring after a neck dissection is a 25% incidence of postoperative shoulder dysfunction among patients who underwent a lateral neck dissection [36], neck numbness or neuropathic pain [37,38], and other postoperative sequelae [36-39]. Further, the increasing use of preoperative ultrasound conceivably decreases the number of clinically node-negative patients [40]. Finally, life-long TSH-suppressive doses of exogenous L-thyroxin after operation are felt to improve the prognosis of patients with occult metastasis. For these reasons, for clinically node-negative patients with PTC, knowledge of the presence or absence of lymphatic metastases may be of only interest in the staging procedure. However, if the SLN examination is negative, it would seem worthwhile not to have to perform further cervical node evaluation. Our results suggest that the dual SLN procedure would best accomplish this.

The previous studies of the efficacy of SLN biopsy in thyroid cancer seldom segregated different subpopulation in different tumor types so as not to produce more focused finding for discrete subpopulation, which may in reality exhibit different biological behaviors. Moreover, we hypothesized that the combination of the blue-dye method and lymphoscintigraphy/gamma probe techniques can have complementary roles and overcome some limits (such as retrieval of SLN outside the central compartment) to increase the possibility of identification of SLN. Thus, our study is, to the best of our knowledge, the largest comparative analysis of two techniques of SLN biopsy for younger female patients with ipsilateral low-risk PTC (T_1-2_N_0_M_0_) in Chinese population. In consistence with previous reports of 21% to 82% in patients without gross evidence of cervical lymph node involvement [15], the incidence in this study was 71.8% in methylene blue group, 60.0% in dual-agent group, and 65.5% in all patients. It demonstrated the feasibility of both procedures of SLN with identification rate of 86.7% for methylene blue only, 100% for dual-agent technique, and 93.3% for all, with accuracy rate of 82.1% for methylene blue only, 93.3% for dual-agent technique, and 88.1% for all (Table 1). This was consistence with the average rate of SLN identification with 83% (range,65%-95%) for blue dye and 96% (range,77%-100%) for ^99 m ^Technetium, and accuracy rate in most patients (range, 80%-100%) [7,8,10-20,35]. A consensus had been reached that using the ^99 m ^Technetium-labeled colloid technique yielded better detection rate of nodal metastases (P = 0.035). Hence, for institutions considering the application of SLN biopsy in low-risk patients with PTC, the combining use of Technetium-99 m for SLN detection offers a potentially higher success rate in identifying SLN. The use of blue dye technique only for SLN detection may be less successful due to transport of the blue dye to the parathyroid glands or prophylactic parathyroid identification resulting the detrimental effect of inevitable perithyroidal tissue dissection and/or minimal thyroid mobilization on the integrity of the lymphatic drainage before injection.

The false-negative rate (FNR) is the single most important quality item for the SLN technique. If a negative SLN biopsy could not definitively exclude the presence of positive basin lymph nodes, it adds no further staging information. High numbers of false-negative SLN would render the specific technique unsuitable for routine practice. In the present study, the FNR of the blue dye technique of SLN is 38.9% and 14.3% for dual-agent technique. The higher FNR of the blue dye technique in my study may due to the small size of the primary tumor and low-risk of patients having more SLN lying outside the central compartment (35.6% outside the central compartment). The FNR of methylene blue only was higher than it of dual-agent technique, though the difference did not reach significance. This may be related to the fairly small number of cases studied. There were little data regarding the FNR for SLN, previous trials reported a FNR of 11.3% for use of Technetium-99 m [20] and 10.0%-17.9%for the blue dye technique [12,13,15,16]. In early-breast cancer, the American Society of Breast Surgeons recommends a rate of SLN identification of 85% with a false-negative rate of 5% or less in order to abandon axillary dissection [29]. Hence, Regardless of the acceptable identification rate of SLN by whichever of the both procedures was adopted, high FNR renders considering further improvement in the SLN technique for this subpopulation before a routine application. It could not exclude the possibility that PTC cells may more likely escape from the first draining lymph node of and metastasize to other lymph nodes (skip metastases) as gastric carcinoma of 20-30% and thoracic esophagus cancer of 50-60%[41-43], because of different patents and biology of lymph node metastasis. More studies should investigate the technical aspects of harvesting the SLN. Novel methods for SLN detection are currently under investigation, as are the use of MR lymphangiography and carbon dye labeling [44]. Furthermore, it still remains uncertain how best to handle the SLN. The intraoperative examination (frozen-section analysis) and the routing pathologic assessment of SLN can underestimate the true incidence of metastasis [43]. False-negative SLNs may contain tiny deposits of microscopic metastatic disease but these may not be caught in the examined histologic slides. Serial sections, immunohistochemical stains [45] and molecular marker assays (based on the reverse transcriptase-polymerase chain reaction technique) increase the detection rate of SLN metastases and could identifying micrometastases [46].

## Conclusion

In our study, comparing to blue dye for younger female patients with ipsilateral low-risk PTC (T_1-2_N_0_M_0_), the combined technique of SLN biopsy could help more accurate lymph node staging and better identification of SLN located out of the central compartment. Regardless of the acceptable identification rate of SLN by whichever of the both procedures was adopted, high FNR renders the SLN technique unsuitable for routing practice; Prophylactic node dissection of level VI might be considered rather than routine practice for patients who did not had SLN biopsy, where only 38.1% of patients had node metastasis.

## Competing interests

There is no conflict of interest that could be perceived as prejudicing the impartiality of the research reported. This work was supported by grants from the National Natural Science Foundation of China (30872377, 81072405), Program for New Century Excellent Talents in University from Ministry of Education of PRC (NCET-08-0486), grant from Zhejiang Provincial Natural Science Foundation of China (R2100528, Y207526) and also sponsored by Zhejiang Provincial Program for the Cultivation of High-level Innovative Health talents.

## Authors' contributions

OH and WLW carried out the conception and design of the study. ZXH participated in the design and administrative support for the study. All authors participated in the provision of patients. OH, WLW and ZXH participated in the data analysis and interpretation of the study, drafted the manuscript. All authors read and approved the final manuscript.

## Pre-publication history

The pre-publication history for this paper can be accessed here:

http://www.biomedcentral.com/1471-2407/11/386/prepub
